# AGMNet: an explainable gated multimodal framework integrating MRI heterogeneity and dopaminergic asymmetry for Parkinson's disease classification

**DOI:** 10.3389/frai.2026.1849408

**Published:** 2026-09-24

**Authors:** T. Gnana Abinaya, R. Sivashankari

**Affiliations:** School of Computer Science Engineering and Information Systems, Vellore Institute of Technology (VIT), Vellore, Tamil Nadu, India

**Keywords:** asymmetry analysis, DAT imaging, gated fusion network, MRI, multi-model deep learning

## Abstract

**Introduction:**

Parkinson's Disease (PD) is a neurological disorder that worsens over time and is marked by interhemispheric asymmetry, dopaminergic degeneration, and structural changes in the brain.

**Methods:**

This paper presents a novel approach: AGMNet- Adaptive Gated Multimodel Network, which is an explainable gated multimodal deep learning framework that combines structural magnetic resonance imaging (MRI), dopamine transporter (DaT) imaging, asymmetry-aware representations, and biomarker features for robust PD classification. The Grad-CAM method is incorporated with the proposed AGMNet framework to improve the model interpretability by identifying image regions that influence classification decisions. The performance of AGMNet framework was evaluated using five-fold cross-validation and compared with established deep learning architectures, including VGG16, VGG19, InceptionV3, and Xception.

**Results:**

The proposed AGMNet has achieved 0.8318 of accuracy, 0.8278 of balanced accuracy, sensitivity of 0.8556, specificity of 0.8000, AUC of 0.711, and Matthews correlation coefficient (MCC) of 0.6458. The proposed AGMNet model demonstrated competitive classification performance relative to the evaluated baseline architectures. The combination of DaT imaging, asymmetry-aware features, and biomarker representations in the proposed model, contributed more strongly to the fused representation than MRI features alone.

**Discussion:**

The findings indicate that adaptive multimodal fusion in the AGMNet framework can improve the integration of complementary structural, functional, asymmetry-related, and biomarker information for PD classification. The learned gate weights further demonstrate the relevance of functional and asymmetry-aware information within the proposed framework, while Grad-CAM provides additional insight into model decision-making. Therefore, the AGMNet framework offers a potentially useful and interpretable framework for multimodal PD classification.

## 1 Introduction

The second most common neurodegenerative disorder is Parkinson disease (PD), which occurs in millions of people throughout the world and puts a significant strain on the medical field. The main characteristic of the illness is progressive loss of dopaminergic neurons in the substantia nigra (Ba and Martin, 2015; Porter et al., 2020), resulting in the manifestations of the disease in both motor (tremor, rigidity, and bradykinesia) and non-motor symptoms. Early and accurate diagnosis of PD is still a serious issue since a clinical assessment is often based on the subjective assessment, The diagnosis of Parkinson's disease usually comes after significant nigrostratial dopaminergic system deterioration. Neurodegenerative process starts year before a clinical diagnosis (Martini et al., 2025) is made, as clinical motor symptoms typically don't show up until there is a substantial loss of dopaminergic neurons and striatal dopamine terminals. The neuroimaging has become a useful modality in augmenting the diagnosis of PD by offering a picture of structural and functional changes in the brain. Cortical and subcortical changes can be assessed by structural magnetic resonance imaging (MRI) and dopamine transporter (DaT) imaging can provide functional information relevant to the dopaminergic activity in the striatum. Notably, PD is also linked to asymmetric degeneration, i.e. with one side of the brain being more involved in the disease than the other; Asymmetry is a clinically relevant imaging characteristic frequently observed in Parkinson's disease and may provide complementary information for disease characterization. In particular, asymmetric reductions in striatal dopamine transporter uptake are commonly reported during DaT-SPECT assessment and reflect the uneven progression of nigrostriatal dopaminergic degeneration (Zarkali et al., 2024; Martini et al., 2025). However, the majority of the current methods use single-modality of data that limits their ability to capture the entire array of PD-related abnormalities. The latest developments in deep learning showed encouraging results in the medical image analysis domain particularly in the convolutional neural networks (CNNs) that have the ability to learn hierarchical features representation automatically. Various models have been used to classify PD using deep learning models like VGG, Inception, and Xception. Although these methods are quite accurate, they have a couple of main limitations namely (i) lack of effective multimodal integration and (ii) low interpretability. Numerous multimodal approaches are based on unaffinity-based simple feature concatenation or averaging approaches that do not ensure that modalities have different weights. In addition, deep learning models can be considered black boxes, which therefore hampers their use in clinical practice because the decision-making process is not transparent. To counter these difficulties, there is increasing demand to have frameworks that can combine the multimodal information in an adaptive manner, and generate interpretable outputs, which are consistent with clinical knowledge. Specifically, the inclusion of asymmetry-aware representations and biomarker-driven features can make a model much more sensitive to PD-specific patterns. Also, explainable artificial intelligence (XAI) algorithms like GradientWeighted Class Activation Mapping (GradCAM) can be used to visualize the contributing regions to model predictions to enhance trust and clinical relevance. In this paper, we present AGMNet (Adaptive Gated Multimodal Network) explainable deep-learning model of Parkinson disease classification that combines MRI, DaT imaging, asymmetry features, and biomarker representations. The suggested model uses feature extractors that are specific to the modality, then uses a gated fusion mechanism, which assigns weights to various modalities dynamically depending on how relevant they are to the classification task. The proposed adaptive gated fusion operation is different from the traditional multimodal fusion methods including early fusion, late fusion, attention-based fusion, and transformer-based fusion, as it is subject-specific instead of fixed or globally learned feature aggregation. Early fusion fuses features from different modalities before learning a modality-specific representation, while late fusion combines modality-specific predictions without taking into account interactions between features. Attention-based and transformer-based fusion methods enhance cross-modal feature interactions; however, they predominantly employ globally learned attention mechanisms without dynamically adapting modality contributions to individual subjects. This adaptive weighting allows the framework to focus on the most informative modalities, suppress less discriminative information to better leverage multimodal feature integration, and give clinically interpretable modality importance during decision making and to improve robustness to inter-subject variability. This approach ensures that the model puts more emphasis on informative characteristics, e.g., dopaminergic activity and asymmetry patterns, and, at the same time, uses structural data of MRI. Moreover, Grad-CAM is added to give visual explanations of the model choices that identify parts of the image that have a clinical meaning both in MRI and DaT images. This study suggests an explainable Adaptive Gated Multimodal Network (AGMNet) for Parkinson's disease classification to overcome the shortcomings of current multimodal fusion techniques, which primarily rely on fixed feature aggregation or globally learned attention mechanisms without subject-specific modality weighting. In contrast to traditional multimodal fusion methods that use fixed aggregation methods, AGMNet adapts the fusion process through training a subject-specific gated fusion mechanism which dynamically learns the relative importance of each modality based on the imaging characteristics of individual patients. This clinically motivated design allows completion of the image information from complementary structural, functional and quantitative imaging and permits a conceptual integration of this information in a way that mimics the diagnostic reasoning used by neurologists. Although several deep learning approaches have explored multimodal fusion for Parkinson's disease classification, most existing methods employ early fusion, late fusion, attention mechanisms, or transformer-based architectures that either concatenate multimodal features directly or assign feature-level attention without explicitly modeling the subject-specific contribution of each modality. Such approaches generally assume fixed or globally learned feature importance, which may not adequately capture the heterogeneous imaging characteristics observed across individual patients. Consequently, an adaptive fusion strategy capable of dynamically weighting each modality according to the characteristics of each subject remains insufficiently explored for multimodal Parkinson's disease diagnosis. This work makes the following principal contributions and introduces novel features:

1. This study proposes a novel framework for explaining multimodal collaborative structural MRI, functional DaT, asymmetry-aware descriptors, and image-derived biomarkers architectures for a unified end-to-end deep learning framework to comprehensively characterize the disease in the context of the classification of Parkinson's disease.

2. The second is an adaptive gated fusion mechanism based on the subject-specific image characteristics of the multimodal images, as opposed to the conventional fixed-weight fusion algorithm. This adaptive fusion allows for individual multimodal representation learning and is a reflection of the clinical diagnostic process in which different weights are placed on the structural, functional, and complementary imaging findings based on the given patient presentation by neurologists.

3. A feature extraction strategy based on asymmetry is directly extracted from multimodal imaging features is introduced in 3. which is motivated by biology. The proposed framework does not employ asymmetry as an individual biomarker, but rather the lateralized information in the images as a complementary descriptor to aid multimodal feature learning and boost Parkinson's disease classification.

4. The combination of quantitative neuroimaging properties and learned feature representations through image-derived biomarker representations with multimodal deep imaging features improves feature discrimination while maintaining clinically relevant imaging information.

5. An explainable artificial intelligence (XAI) framework using Grad-CAM is added to make the model more interpretable and transparent and to increase the confidence of clinicians in the model's clinical decision making by providing a visual explanation of the disease-relevant imaging regions.

6. The comprehensive experimental evaluation proves that AGMNet is consistently better than existing state-of-the-art single-modality and multimodal deep learning models, and the adaptive gating analysis offers clinically relevant information about the importance of each modality for the individual patient, thus showcasing the potential of the proposed framework as an explainable clinical decision-support system for Parkinson's disease evaluation.

The remaining portions of this work are organized as follows. Relevant research on Section 2 summarizes relevant research on multimodal learning and PD categorization. The suggested AGMNet framework provides a detailed description in Section 3. The experimental setup and findings are shown in Section 4. The results and clinical implications are covered in Section 5, and the paper's future directions are discussed in Section 6.

## 2 Related work

The use of machine learning (ML) and deep learning (DL) methods has contributed to the automated diagnosis of Parkinson's disease (PD) significantly, especially on the processing of neuroimaging and clinical data. Early research focused on classical ML models such as Support Vector Machines (SVM), Random Forests (RF), and logistic regression using manually engineered features derived either out of MRI or clinical data (Ozcift, 2012; Lamba et al., 2022; Ba and Martin, 2015; Devadas and Hiremani, 2022), with traditional feature engineering methods despite their reasonable performance, these methods were limited by low scalability and the necessity to use feature engineering and poor results with high-dimensional and heterogeneous data.

The most popular approach of PD detection using neuroimaging data is now convolutional Neural Networks (CNNs) with the introduction of deep learning. They have also applied pre-trained networks, such as AlexNet, ResNet, DenseNet, and EfficientNet, to MRI-based classification and have achieved superior results compared to standard methods (Sivaranjini and Sujatha, 2020; Zhao et al., 2022; Abbas et al., 2025; Safai et al., 2022), in which In fact, CNN-based models trained on MRI images have been said to achieve accuracy of approximately between 88 to 96 percent which highlights the potential of deep networks to identify subtle structural differences in the brain that are associated with PD. However, the models are predominantly unimodal, as they do not reveal the multifacetedness of PD, since they utilize only imaging data.

To compensate what the unimodal models are not, the recent researches have been lapsing into multimodal learning that encompasses the use of imaging information with clinical and behavioral information. Dentamaro et al. (2024) have created a deep learning multimodal framework, which inter-polyphases MRI data with clinical characteristics, using a joint co-learning strategy and an Excitation Network (EN) (Hu et al., 2023), and exhibits better feature interaction between modalities. They demonstrated, through their methodology, that multimodal fusion is a significant improvement in diagnostic accuracy with the F1-score of 96.5% on DenseNet+EN. Intelligible artificial intelligence (XAI) models such as Integrated Gradients and attention heatmap were also applied to reveal clinically meaningful brain regions (Mozhdehfarahbakhsh et al., 2021; Magesh et al., 2020), enhancing the interpretability of deep models.

Multimodal CNN-PD uses a ResNet backbone, a Meta-Guided Cross-Attention (MGCA) mechanism, and Convolutional Block Attention modules (CBAM) (Zhi et al., 2026; Palakayala et al., 2025; Pianpanit et al., 2021) to integrate MRI with clinical metadata such as motor scores, demographics, and genetic biomarkers to improve cross-modal feature learning. This model achieved an accuracy of 95.68 multiclass classification and was strongly generalized to external data.

In addition to the MRI based, several studies have been performed in relation to incorporation of multiple modalities, including speech signals, gait and handwriting patterns and biosignals, EEG, and SPECT (Keserwani et al., 2024; Frasca et al., 2025; Kibtia et al., 2020), and the role of heterogeneous data fusion. The diagnostic accuracy of multimodal models that combine these heterogeneous data sources has been shown to be higher than 94 or even higher as compared to single-modality systems.

Irrespective of these developments, the current multimodal models have a number of critical weaknesses. Firstly, most of the studies are primarily interested in MRI being used in conjunction with clinical or behavioral data, but functional imaging methods that directly indicate dopaminergic degeneration such as Dopamine Transporter (DaT) imaging are not actively used (Porter et al., 2020; Vijayakumari et al., 2023). Second, even though attentional mechanisms have been popular, such as CBAM, and cross-attention, they lack mechanisms of adaptive gating that can dynamically control the input of either modality in various conditions. Hemispheric asymmetry has been investigated in clinical and imaging-based studies using region-wise or statistical measurements (Wang et al., 2021). Nevertheless, its incorporation as a learnable element in multimodal deep learning frameworks is still restricted, especially in systems that simultaneously model asymmetry-aware, structural, and functional representations. Fourth, XAI methods are gaining more and more popularity, but they are not always multimodal and offer no holistic multimodal interpretability framework (Nazari et al., 2022; Aggarwal et al., 2024; Teng et al., 2025).

Moreover, heterogeneity of data, smaller size of data set, and their extrapolation to other clinical settings remain significant drawbacks. Multimodal models are not easy to define fine-grained interactions between structural and functional features, which result in the suboptimal combination of complementary information. Moreover, lightweight and computationally efficient architectures are not available and this limits real-time clinical deployment.

To overcome these weaknesses, this study suggests an Explainable Adaptive Gated Multimodal Network (AGMNet) which combines MRI heterogeneity, DaT uptake, and asymmetry biomarkers in one deep learning framework. On the one hand, unlike the existing approaches, AGMNet is a gated fusion mechanism that dynamically regulates the contribution of each modality that enables powerful integration of features and improved classification performance. In addition, the integration of features that are sensitive to asymmetry promotes the capability of the model to reflect clinically significant PD progression. The provided framework is also based on explainable AI practices that provide a clear understanding of structural and functional biomarkers that improve clinical reliability and trust.

## 3 Materials and methods

### 3.1 Dataset description

This study used the publicly available NTUA Parkinson dataset from the Kaggle website. The data includes subject-level multimodal brain imaging data, including structural magnetic resonance imaging (MRI) and dopamine transporter (DaT) scans. The subjects are labeled as either Parkinson's disease (PD) or healthy controls (HC).

We only considered those subjects who have both MRI and DaT modalities to ensure multimodal learning. This guarantees that each sample provides both structural and functional information for training the model. In this paper, we used the publicly available NTUA Parkinson Dataset on Kaggle, comprising of multimodal neuroimaging (MRI and DaT scans) of 78 subjects (55 with Parkinson's disease (PD) and 23 healthy controls). The dataset comprised of 43,087 MRI images and 920 DaT images that were utilized in the training, validation, and testing of the proposed AGMNet model. Despite offering synchronized MRI and DaT imaging appropriate for multimodal learning, the NTUA Parkinson dataset's comparatively small cohort of 78 individuals is a significant drawback. Small datasets may not adequately reflect the heterogeneity in illness characteristics across larger clinical populations and may raise the risk of overfitting. As a result, the study's findings should be seen as a preliminary validation of the suggested AGMNet framework; additional testing on bigger and more varied cohorts is required to determine its clinical generalizability and robustness. MRI images present structural brain data whereas DaT scans give information of dopaminergic activity in the striatum, both of which are crucial in identifying the effect of Parkinson disease in the brain.These multimodal neuroimaging features can be used to have a complete classification model incorporating both structural and functional brain data.

The NTUA Parkinson dataset was accessed from the publicly available Kaggle repository and used as the primary dataset for this study (Vaghasiya, 2023). The dataset is appropriate for analyzing the suggested adaptive gated fusion technique and determining the contribution of asymmetry-aware imaging descriptors to Parkinson's disease classification due to the availability of subject-level multimodal imaging data whereas bigger cohorts like the Parkinson's Progression Markers Initiative (PPMI) give comprehensive clinical and imaging information. The availability of synchronized structural and functional imaging modalities was regarded as a crucial factor for dataset selection because the main goal of this study is to assess the efficacy of multimodal picture fusion and adaptive gating mechanisms. Future research will use bigger independent cohorts, such as PPMI, to examine the generalizability of the suggested AGMNet framework.

Images for both MRI and DaT scans were resampled to a standard resolution of 128 × 128 and grayscaled. We used intensity normalization to scale the data across individuals and scans.

We used 5-fold subject-level cross-validation for model evaluation. Each fold consisted of about 80% of the data for training and 20% for testing, with a stratified sampling strategy to maintain class balance between PD and controls. In the training set, 10% of the subjects were used as a validation set for tracking the learning process and avoiding overfitting.

We used a subject-wise stratified sampling approach to avoid data leakage, ensuring that images from the same subject are not included in both training and testing sets.This allows for a more accurate assessment of model performance on new subjects. The dataset is publicly accessible and has been deidentified, adhering to ethical guidelines.

### 3.2 Extraction of biomarkers and descriptors

Besides deep feature representations of MRI and DaT images, we also compute quantitative image-related descriptors that convey significant clinical characteristics of Parkinson disease. These descriptors are computed directly based on the imaging data (MRI and DaT) and do not depend on some external clinical biomarkers, like blood tests or other medical measurements. These descriptors are instead created based on image intensities and structural patterns perceived in the images. The descriptors include:

1. MRI-based heterogeneity measures: These measures are the measures of variability of intensities distribution of the brain tissues. Simply put, this measure is an indicator of the variation between various regions of the brain in regard to tissue properties (Santa Cruz et al., 2023), which may reflect alterations in relation to the Parkinson disease.

2. DaT-based uptake measures: Measures of uptake are used to determine the level of dopamine activity in the striatum, which is a part of the brain that is usually impacted by Parkinson disease. We determine this by assessing the intensity of the DaT images, which are the amount of dopamine uptake in the striatum (Santa Cruz et al., 2023).

3. Asymmetry measurements: Parkinson disease usually causes lateralized motor symptoms (Yang et al., 2021) i.e., the disease can have a greater impact on one side of the body compared to the other. The asymmetric measures are founded on the differences in left-right intensity of the DaT images which represent the inequalities in the brain functions of the two hemispheres that is a typical aspect of Parkinson.

To measure the structural abnormalities, the heterogeneity features of the MRI are calculated in terms of statistical measures: variance and entropy (Santa Cruz et al., 2023). These tests portray the differences in the brain structure -they assist in determining the areas that exhibit abnormality in the form of tissue intensity, which may be an indication that the individual has Parkinson disease.

DaT imaging provides an indirect assessment of presynaptic dopaminergic function through the evaluation of dopamine transporter availability within the striatum. Reduced tracer uptake is frequently employed as a supporting imaging marker in the evaluation of Parkinsonian disorders and is typically linked to degeneration of nigrostriatal dopaminergic pathways (Tolosa et al., 2021). This uptake of DaT is then extracted to obtain related features which show the amount of dopamine activity that is still present in the brain.

Lastly, the asymmetry indices are achieved by computing the normalized difference in left-right intensity of the DaT images. These measures are the degree of imbalance between the left and right hemispheres of the brain and are indicative of the asymmetry seen in patients of the Parkinson disease.

These descriptors are not based on anatomical division of the brain which may need time-consuming and costly manual annotation. They are instead computed directly on the images based on the intensity values and the process is more efficient and scalable.

These descriptors are combined into one descriptor vector and then converted to a latent space and fed to the multimodal gated fusion model to make a classification.

### 3.3 Data preprocessing

MRI and DaT images were preprocessed before model training to guarantee consistency and enhance feature extraction. To guarantee consistent input dimensions among participants, each image was scaled to a set spatial resolution.

Grayscale MRI and DaT images were initially scaled to 128 × 128 pixels for the proposed AGMNet model. However, for compatibility with common CNN-based architectures like VGG16 and InceptionV3, the images were resized to 299 × 299 pixels as required by these baseline models. The resizing ensures that the images meet the input size requirements for the CNN models and allows for effective learning of relevant features. After resizing, the images are processed by the respective feature extraction models, and the final size for AGMNet is 256 × 256 pixels, used in the multimodal fusion network. This process ensures consistency and interoperability across different models.

The intensity values of pixels were scaled to a constant range so as to minimize changes that occurred due to varying imaging conditions and acquisition parameters. This normalization stage enhances better convergence and model stability throughout training.

Data augmentation was implemented to the training set to improve model's generalization and reduce overfitting. These were random horizontal flipping, rotation in a restricted range of angles and slight variations in intensity. These changes make medical imaging data vary as in the real world and enhance robustness.

In addition, both MRI and DaT modalities had the same amount of preprocessing to ensure that structural and functional representations were in line. The preprocessing pipeline will be used to make sure the input data are fit to undergo multimodal feature extraction and fusion in the proposed framework.

## 4 Proposed approach: AGMNet architecture

This study suggests AGMNet (Adaptive Gated Multimodal Network) which is an explainable deep learning model capable of incorporating structural MRI, functional DaT, and image-derived asymmetry-aware quantiative features for Parkinson disease classification. The AGMNet architecture proposed (see Table 1) combines two forms of multimodal neuroimaging data, including MRI and DaT images to classify Parkinson disease. The images are firstly shrunk to 128 × 128 pixels then fed to two distinct feature extractors namely the MRI encoder and the DaT encoder. These two encoders are multiple convolutional layers with batch normalization and ReLU activation and max-pooling (Table 1), which enable the network to learn hierarchical features of the images. Both encoders produce a 64-dimensional feature vector. These features are in turn subjected to a set of convolutional blocks (Table 1), each of which seeks to enhance the feature maps. The network uses adaptive average pooling to reduce the feature maps to a 64-dimensional feature, which implies that the features extracted can be used in the subsequent steps of the model. One of the crucial issues of the architecture is the asymmetry branch that concentrates on the difference between the left and right hemispheres in the feature map (Table 1). This unbalanced nature is a well-established characteristic of the Parkinson disease, in which one side of the brain is usually more impaired than the other. The asymmetry characteristics are represented and analyzed by a fully connected layer, which results in a 32-dimensional vector that represents this imbalance. These different feature streams (MRI, DaT, and asymmetry features) are then combined in the descriptor branch, summarized in Table 1. This branch produces a 16-dimensional descriptor vector, which combines the most interesting features of each modality, such as structural, functional and asymmetry-related data. The gate network regulates the integration of these features (Table 1). This network involves the adaptive control of the contribution of each stream of features by using learnable weights so the model can focus on the most relevant features to use in the classification process. Such weights are obtained by training a series of fully connected layers using a sigmoid activation function that ensures that most significant features are highlighted in the process of training. Lastly, all the fused features are fused by using a weighted linear fusion layer (Table 1) to combine them into a single feature. This vector is then subjected to fully connected layers resulting in a 32 dimensional vector which is inputted into the last classifier. The classifier is implemented with the ReLU activation, dropout regularization and a sigmoid activation at the final layer in order to generate a binary classification score that defines whether the input is a Parkinson disease or a healthy control.

**Table 1:** AGMNet architecture configuration.

Component	Configuration	Output dimension
MRI input	Grayscale MRI image	1 × 128 × 128
DaT input	Grayscale DaT image	1 × 128 × 128
MRI encoder	Three CNN blocks: Conv2D, BatchNorm, ReLU, MaxPool	64-d vector
DaT encoder	Three CNN blocks: Conv2D, BatchNorm, ReLU, MaxPool	64-d vector
CNN Block 1	3 × 3 Conv, 16 filters, MaxPool 2 × 2	16 × 64 × 64
CNN Block 2	3 × 3 Conv, 32 filters, MaxPool 2 × 2	32 × 32 × 32
CNN Block 3	3 × 3 Conv, 64 filters, MaxPool 2 × 2	64 × 16 × 16
Global pooling	Adaptive average pooling	64
MRI/DaT FC	Fully connected layer	64
Asymmetry branch	Left–right feature-map difference + FC	32
Descriptor branch	[*H*_MRI_, *U*_log_, *A*] + FC	16
Gate network	FC (176,64) + ReLU + FC (64,4) + Sigmoid	4 weights
Projection layers	All features projected to common feature space	64 each
Fusion	Weighted linear fusion	64
Classifier	FC (64,64) + ReLU + Dropout (0.3) + FC (64,32)	32
Output layer	FC (32,1) + Sigmoid	Binary output

The proposed architecture as shown in Figure 1 is designed to effectively capture complementary information from multiple modalities while maintaining interpretability. Multimodal feature extraction, asymmetry modeling, gated feature fusion, and classification are the four main parts of the entire pipeline.

**Figure 1:**
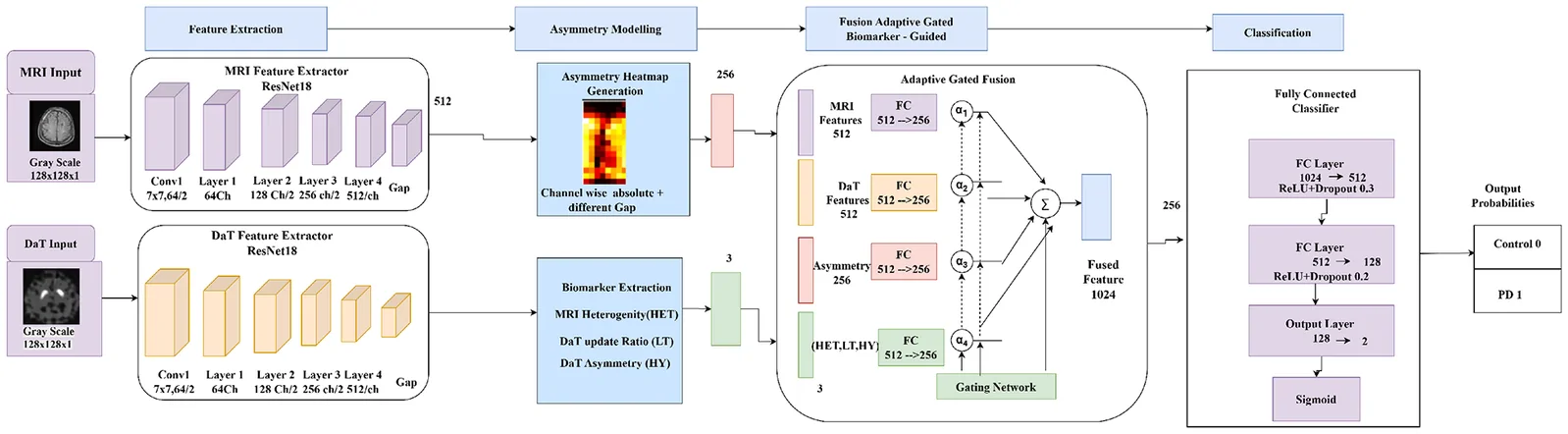
Architecture of the proposed AGMNet framework for PD classification.

### 4.0.1 Multimodal feature extraction

MRI and DaT modalities are inputted into two convolutional feature extractors. The feature extractors are all convolutional layers, which have batch normalization, ReLU activation, and max-pooling to extract hierarchical features. The layers allow the model to learn the low-level to high-level feature representations of both the MRI and DaT images. The features are flattened and this is followed by feeding into fully connected layers to produce feature embeddings, which are a summary of the most significant information of each modality.

### 4.0.2 Asymmetry modeling

The asymmetry features, capturing hemispheric asymmetries seen in the Parkinson disease, and the image-based descriptor vector (both of MRI and DaT images) are also projected to the same latent feature space by fully connected layers. This makes sure that feature streams (say, of the MRI, the DaT or the asymmetry information) all lie in a common dimensional feature space. The network can better integrate the various forms of information to be classified because it can convert all the features into one space.

### 4.0.3 Gated feature fusion

An adaptive gating mechanism is used to fuse the multimodal feature streams. Here, a sigmoid is used to apply learnable weights to each modality. These weights can be used to make the model adaptively give more importance to the most relevant features, by weighting each modality by importance in classification. The gating mechanism is used to provide the model greater flexibility and allows it to dynamically focus on the most informative aspects of the MRI, DaT, and asymmetry branches in the process of the fusion.

### 4.0.4 Classification

Lastly, binary classification (Parkinson disease vs. control) is done using the fused features passed through completely connected layers. The gating mechanism which allows the architecture to adaptively weight feature contributions makes sure that the final classification decision is influenced by the most pertinent information obtained out of the multimodal neuroimaging data.

AGMNet has the following architecture that involves the following stages.

### 4.1 Multimodal feature extraction

Considering input MRI and DaT images, feature extractors are used to extract structural and functional information on a modality-specific scale:


$$\begin{array}{l} F_{\text{MRI}}=f_{p_{1}}\left(Y_{\text{MRI}}\right) \end{array}$$ (1)

where *F*_MRI_ represents the extracted MRI features from the processed input *Y*_MRI_ *f*_*p*_1__(·) is a learnable feature extraction function as seen in Equation 1.


$$\begin{array}{l} F_{\text{DaT}}=f_{p_{2}}\left(Y_{\text{DaT}}\right) \end{array}$$ (2)

where *f*_*p*_1__ *f*_*p*_2__ are learnable parameters as seen in Equation 2.

### 4.2 Asymmetry-aware feature modeling

Asymmetric degeneration in both hemispheres of the brain is a hallmark of Parkinson's disease. Asymmetry features are calculated as follows in order to capture this clinically relevant pattern:


$$\begin{array}{l} A=\frac{\left|I_{L}-I_{R}\right|}{I_{L}+I_{R}+\epsilon } \end{array}$$ (3)

where ϵ is a tiny constant to guarantee numerical stability and *I*_*L*_ and *I*_*R*_ are the mean intensity values taken from the left and right sides of the DaT image, respectively as seen in Equation 3. This approach uses normalized intensity differences to capture inter-hemispheric imbalance. The proposed asymmetry descriptor is meant to measure the hemispherical intensity pattern differences in DaT images. From a clinical standpoint, clinically evident Parkinsonian degeneration often coincides with an unequal loss of dopamine transporter uptake, specifically in the striatal area. This image-derived metric, therefore, was added to account for the intensity discrepancies between the left and right sides, which could be relevant to Parkinsonian changes, but maintain consistency with the data available.

Instead of using clinically established striatal binding ratios (SBR), the suggested asymmetry formulation is based on intensity differences derived from images. The lack of anatomical labeling and region-wise segmentation in the dataset is the driving force behind this design decision. Rather, a data-driven method is used, in which DaT pictures are used to compute left-right intensity differences.

Although SBR-based asymmetry measurements are frequently employed in clinical research, the current dataset lacks detailed region-of-interest definition (such as striatum and reference areas). To ensure consistency with the available data while preserving computational simplicity, a normalized intensity-based asymmetry metric is used.

### 4.3 Quantitative descriptor extraction derived from images

To capture structural variability, functional uptake variation, and inter-hemispheric asymmetry, a collection of image-derived quantitative descriptors is generated from MRI and DaT images in addition to deep feature representations. These descriptors do not reflect external clinical or biochemical biomarkers; instead, they are directly determined from imaging intensities.

#### 4.3.1 MRI heterogeneity

The ratio of intensity variation within a region of interest is used to quantify the structural heterogeneity of MRI:


$$\begin{array}{l} H_{\text{MRI}}=\frac{\text{std}\left(R_{\text{MRI}}\right)}{\text{mean}\left(R_{\text{MRI}}\right)+\epsilon } \end{array}$$ (4)

where ϵ is a small constant to prevent division by zero and *R*_MRI_ represents the chosen MRI region of interest. This measure measures intensity dispersion in relation to the mean, as seen in Equation 4.

#### 4.3.2 DaT uptake ratio

The left and right area intensities in relation to a reference region are used to calculate the functional uptake characteristics using DaT imaging:


$$\begin{array}{l} U_{L}=\frac{I_{L}}{I_{\text{ref}}+\epsilon },\;U_{R}=\frac{I_{R}}{I_{\text{ref}}+\epsilon } \end{array}$$ (5)

The average uptake ratio is then computed as:


$$\begin{array}{l} U_{\text{avg}}=\frac{U_{L}+U_{R}}{2} \end{array}$$ (6)

The dynamic range is stabilized by using a logarithmic transformation:


$$\begin{array}{l} U_{\log}=\log\left(1+U_{\text{avg}}\right) \end{array}$$ (7)

As indicated in Equations 5–7, these steps normalize and compress the uptake variation.

#### 4.3.3 Asymmetry index

The normalized difference between the left and right uptake levels is used to calculate inter-hemispheric asymmetry:


$$\begin{array}{l} A=\frac{\left|U_{L}-U_{R}\right|}{U_{L}+U_{R}+\epsilon } \end{array}$$ (8)

The degree of imbalance between the two hemispheres is captured by this index, as described in Equation 8.

#### 4.3.4 Descriptor vector

The final descriptor vector is formed as:


$$\begin{array}{l} B=\left[H_{\text{MRI}},\;U_{\log},\;A\right] \end{array}$$ (9)

The image-derived quantitative descriptor set is represented by the vector *B* in Equation 9, which is integrated into the multimodal fusion procedure.

### 4.4 Multimodal fusion with gates

AGMNet uses an adaptive gated fusion approach to efficiently integrate data from several modalities. The model learns modality-specific weights rather than just concatenation as in Equation 10:


$$\begin{array}{l} F_{fused}=\overset{4}{\underset{i=1}{\sum }}\alpha_{i}F_{i} \end{array}$$ (10)

where *F*_*i*_∈{*F*_*MRI*_, *F*_*DaT*_, *F*_*asym*_, *F*_*bio*_} denote structural, functional, asymmetry-aware, and image-derived descriptor features, respectively.MRI heterogeneity, DaT uptake, and asymmetry indices are among the quantitative parameters produced from the input images that make up the descriptor vector *F*_*bio*_. The proposed adaptive gated fusion mechanism has been different from the conventional multimodal fusion strategies as it explicitly learns the importance of the modalities from the subject instead of fixed feature aggregation. Early fusion is a heterogeneous approach that fuses features after only a limited amount of modality-specific representation learning through a simple concatenation, while late fusion is a homogeneous approach that fuses predictions obtained from the independent processing of the modalities without modeling any feature-level interaction. Attention- or transformer-based fusion is able to capture more interactions between cross-modal features by learning attention weights between features, but it typically does not explicitly estimate the diagnostic role of each imaging modality for each subject. The proposed gating module, in contrast, dynamically assigns adaptive weights to MRI, DaT imaging and asymmetry-aware descriptors and biomarker features, depending on their contribution for each subject. This modality-specific weighting helps to amplify the most informative modalities and damp down less discriminative information, thereby improving multimodal feature integration and interpretability for AGMNet.

### 4.5 Layer of classification

A completely connected classification head receives the fused feature representation. At the output layer, a sigmoid activation function is used to produce a probability score for binary classification (control vs. Parkinson's disease). In order to provide stable optimization and probabilistic interpretability, the model is trained using binary cross-entropy with logits.

### 4.6 Grad-CAM explainability

To enhance interpretability, regions that contribute to model predictions are represented using Gradient-weighted Class Activation Mapping (Grad-CAM). This makes it possible to identify clinically significant regions like the striatum in DaT pictures which reflects dopaminergic activity, and cortical areas in MRI studies. Explainability is incorporated to guarantee that the model's choices are understandable and consistent with established neurological patterns.


$$\begin{array}{l} L_{\text{Grad}}^{c}=\text{ReLU}\left(\underset{i}{\sum }\beta_{k}^{c}A^{k}\right) \end{array}$$ (11)

where $L_{\text{Grad}}^{c}$ is the class-specific localization map, $\beta_{k}^{c}$ are the weights, and *A*^*k*^ are the feature maps as seen in Equation 11.

### 4.7 Training details

The AGMNet model was implemented in a supervised deep learning binary classification framework (PD vs. Control). The Adam optimizer, with adaptive learning rates, was used for parameter optimization during training, and the output of the model was passed through a sigmoid function to get the final classification probabilities with logits.

The optimization objective (loss function) used was binary cross-entropy, which is appropriate for probabilistic binary classification problems and facilitates efficient parameter optimization.

The model was trained for a fixed number of epochs with a fixed batch size, without employing early stopping. To prevent overfitting, dropout regularization was used in the fully connected layers with dropout rates of 0.2 and 0.3.

A random seed (42) was set to maintain the consistency of cross-validation and model initialization.

5-fold cross-validation was employed to ensure a fair and reliable evaluation of the model. The data was divided into five disjoint sets at the level of individuals. Four of these subsets were used for training, and one for testing. For each fold, the training set was further divided into a training subset (90%) and a validation subset (10%).

This process was repeated for each fold, and the metrics such as accuracy, balanced accuracy, Area Under the Curve (AUC), Matthews Correlation Coefficient (MCC), sensitivity and specificity were calculated for each fold. The results were averaged over all the folds, and reported as the mean and standard deviation.

As shown in Table 2, the model was configured with specific parameters, such as a batch size of 8, a learning rate of 1 × 10^−3^, and dropout rates of 0.2 and 0.3, to prevent overfitting. Accuracy Classification accuracy measures the proportion of correctly classified samples among all evaluated samples and is computed as Equation 12

**Table 2:** Training and hyperparameter configuration of the proposed AGMNet model.

Parameter	Value
Input image size (AGMNet)	128 × 128 (grayscale)
Train–test split	80% : 20% (subject-wise stratified)
Batch size	8
Number of epochs	20
Optimizer	Adam
Learning rate	1 × 10^−3^
Loss function	Binary cross-entropy
Dropout rates	0.2, 0.3
Weight initialization	Default (PyTorch)
Random seed	42
Evaluation metrics	Accuracy, balanced accuracy, AUC, MCC, sensitivity, specificity

Classification accuracy is computed as

$$\begin{array}{l} Accuracy=\frac{TP+TN}{TP+TN+FP+FN} \end{array}$$ (12)

where TP, TN, FP, and FN denote the number of true positives, true negatives, false positives, and false negatives, respectively. Balanced accuracy is computed as Balanced accuracy is calculated as the arithmetic mean of sensitivity and specificity Equation 13:

$$\begin{array}{l} Balanced\;Accuracy=\frac{\text{Sensitivity}+\text{Specificity}}{2} \end{array}$$ (13)

Balanced accuracy provides an unbiased measure of classification performance by equally considering the model's ability to correctly identify both positive and negative classes. Unlike conventional accuracy, it is less sensitive to class imbalance and is therefore widely adopted for evaluating medical diagnostic models, where unequal class distributions are common.

## 5 Results

### 5.1 Model performance comparison

The number of baseline models, VGG16, VGG19, InceptionV3, and Xception, were used to assess to evaluate the performance of the proposed AGMNet model. Accuracy, balanced accuracy, AUC, MCC, sensitivity, and specificity were used in the assessment. Predicted probabilities were transformed into binary class labels using a decision threshold of 0.5 to calculate accuracy, sensitivity, specificity, and MCC. The performance of the proposed AGMNet are summarized in Table 3 baseline networks under 5-fold cross-validation. AGMNet achieves the best accuracy (0.8318 ± 0.0563) and MCC (0.6458 ± 0.1195), suggesting a high agreement between predictions and ground truth, as well as balanced performance across both classes. AGMNet's balanced accuracy (0.8278 ± 0.0838) is comparable to the top-performing baseline, InceptionV3 (0.8417 ± 0.0543), indicating that AGMNet is robust to class imbalance.

**Table 3:** Performance comparison of models using 5-fold cross-validation (mean ± standard deviation).

Model	**Acc**.	**Baln Acc**.	**AUC**	MCC
**AGMNet (proposed)**	**0.8318** **±0.0563**	0.8278 ± 0.0838	0.7579 ± 0.1253	**0.6458** **±0.1195**
InceptionV3	0.7985 ± 0.0716	**0.8417** **±0.0543**	**0.8076** **±0.0720**	0.6415 ± 0.0957
VGG16	0.6621 ± 0.0781	0.7569 ± 0.0553	0.6954 ± 0.1800	0.4946 ± 0.0862
VGG19	0.7136 ± 0.1364	0.7111 ± 0.1026	0.6194 ± 0.1842	0.4555 ± 0.1479
Xception	0.7803 ± 0.0418	0.8292 ± 0.0272	0.7657 ± 0.0411	0.6137 ± 0.0362

Bold Values correspond to highest metric value for each models.

Table 3 evaluates InceptionV3 has the best AUC (0.8076 ± 0.0720) in terms of discriminative abilities, followed by Xception and AGMNet. AGMNet exhibits competitive performance (0.7579 ± 0.1253) with rather consistent fluctuation among folds, but not achieving the greatest AUC. This suggests that the suggested model performs better in correlation-based measures like MCC while maintaining consistent discriminative capability.

The findings indicate that AGMNet provides a good compromise between discriminative power and correlation-based metrics (MCC) with respect to classification accuracy.Even though InceptionV3 obtains the highest ROC-AUC (0.8076 ± 0.0720) and slightly higher balanced accuracy (0.8417 ± 0.0543), AGMNet is regarded to be the superior one as it consistently outperforms InceptionV3 on various clinically relevant evaluation metrics. Specifically, AGMNet showed the highest Classification Accuracy (0.8318 ± 0.0563), Sensitivity (0.8556 ± 0.0985), and MCC (0.6458 ± 0.1195), which means that it is more consistent with the classification class label and has a better detection ability for Parkinson's disease. Moreover, AGMNet is designed to fuse complementary structural MRI, DaT imaging and quantitative biomarker descriptors via the proposed adaptive gated fusion mechanism, in contrast to InceptionV3 which is based on a traditional single-modality CNN. The fusion strategy in this research is specific to the modality and allows the model to focus on the most informative modality at the right time. AGMNet is a multimodal feature integration and adaptive fusion framework with high sensitivity and enhanced interpretability, which has a more comprehensive and clinically applicable diagnostic capability, though the ranking performance, including the ROC-AUC and balanced accuracy, is slightly better in InceptionV3. Table 4 evaluates the clinical performance of the proposed model in terms of sensitivity and specificity, which are important diagnostic criteria. Sensitivity measures the model's accuracy in detecting cases of Parkinson's disease (PD), while specificity measures its accuracy in detecting controls.

**Table 4:** Sensitivity and specificity comparison using 5-fold cross-validation (mean ± standard deviation).

**Model**	Sensitivity	Specificity
**AGMNet (proposed)**	**0.8556** **±0.0985**	0.8000 ± 0.2092
InceptionV3	0.7333 ± 0.1304	0.9500 ± 0.1118
VGG16	0.5139 ± 0.1107	**1.0000** **±0.0000**
VGG19	0.7056 ± 0.2892	0.7167 ± 0.2981
Xception	0.7083 ± 0.1062	0.9500 ± 0.1118

Bold Values correspond to highest metric value for each models.

The proposed AGMNet has the highest sensitivity (0.8556 ± 0.0985), indicating a high ability to identify PD cases and avoid false negatives. This is crucial for clinical applications, as false negatives may result in delayed diagnosis and progression of disease. On the other hand, VGG16 reaches 100% specificity (1.0000 ± 0.0000), meaning no false positives; but with lower sensitivity (0.5139 ± 0.1107), indicating that the model is biased toward the negative class and less capable of detecting PD cases.

InceptionV3 and Xception have high specificity (0.9500 ± 0.1118) but lower sensitivity, suggesting that they are conservative in their predictions. The proposed AGMNet strikes a better balance of specificity (0.8000 ± 0.2092) and sensitivity, ensuring that the disease is detected while maintaining reasonable reliability. This is desirable from a clinical perspective as it allows robust disease detection without significantly rising the number of false positives.

In summary, the findings suggest AGMNet offers a clinically relevant performance trade-off, with an emphasis on early and accurate diagnosis of PD and acceptable diagnostic performance.

### 5.2 Confidence interval analysis

For further validation of the robustness of the proposed framework, 95% bootstrap confidence intervals were calculated for all the major evaluation metrics used, including accuracy, balanced accuracy, ROC-AUC, MCC, sensitivity, and specificity metrics, as detailed in Table 5. The confidence intervals reflect the spread of the estimated performance and give an idea of the reliability of the results when the bootstrap is repeated. AGMNet achieved an accuracy of 0.8315 (95% CI: 0.7288–0.9153), balanced accuracy of 0.8167 (95% CI: 0.6989–0.9212), MCC of 0.6160 (95% CI: 0.3870–0.8144), and sensitivity of 0.8555 (95% CI: 0.7391–0.9512), demonstrating stable and consistent diagnostic performance. While the proposed adaptive gated fusion mechanism and its multimodal feature integration and higher interpretability of the model were significant advantages for the overall classification accuracy, the overall model interpretability and the multimodal feature integration were also better than those of InceptionV3, albeit with slightly wider confidence intervals for all the evaluation metrics. The results suggest good performance and consistency of AGMNet when using bootstrap resampling.

**Table 5:** Performance comparison of the evaluated models with 95% bootstrap confidence intervals.

**Model**	**Accuracy**	**Baln acc**.	**AUC**	MCC	Sensitivity	Specificity
AGMNet (proposed)	0.8315 (0.728–0.915)	0.8167 (0.698–0.921)	0.7127 (0.559–0.852)	0.6160 (0.387–0.814)	0.8555 (0.739–0.951)	0.7779 (0.571–0.950)
InceptionV3	0.7966 (0.694–0.898)	0.8379 (0.745–0.920)	0.6993 (0.556–0.834)	0.6230 (0.438–0.791)	0.7311 (0.594–0.866)	0.9448 (0.818–1.000)
VGG16	0.6629 (0.542–0.779)	0.7571 (0.678–0.837)	0.5927 (0.431–0.748)	0.4931 (0.356–0.635)	0.5142 (0.357–0.674)	1.0000 (1.000–1.000)
VGG19	0.7123 (0.593–0.830)	0.7156 (0.584–0.834)	0.6188 (0.452–0.773)	0.4013 (0.150–0.632)	0.7070 (0.565–0.840)	0.7241 (0.500–0.923)
Xception	0.7809 (0.678–0.881)	0.8269 (0.734–0.909)	0.7134 (0.581–0.836)	0.6017 (0.422–0.768)	0.7086 (0.571–0.840)	0.9452 (0.823–1.00)

### 5.3 ROC curve analysis

The ROC curves of all models are presented in Figure 2, which were obtained by applying their predicted probability on different classification thresholds. The calculated AUC values demonstrate similar discriminatory capability of the deep learning models. In particular, AGMNet has an AUC of 0.711, similar to that of Xception (0.713) and InceptionV3 (0.702), and superior to VGG16 (0.592) and VGG19 (0.619).

**Figure 2:**
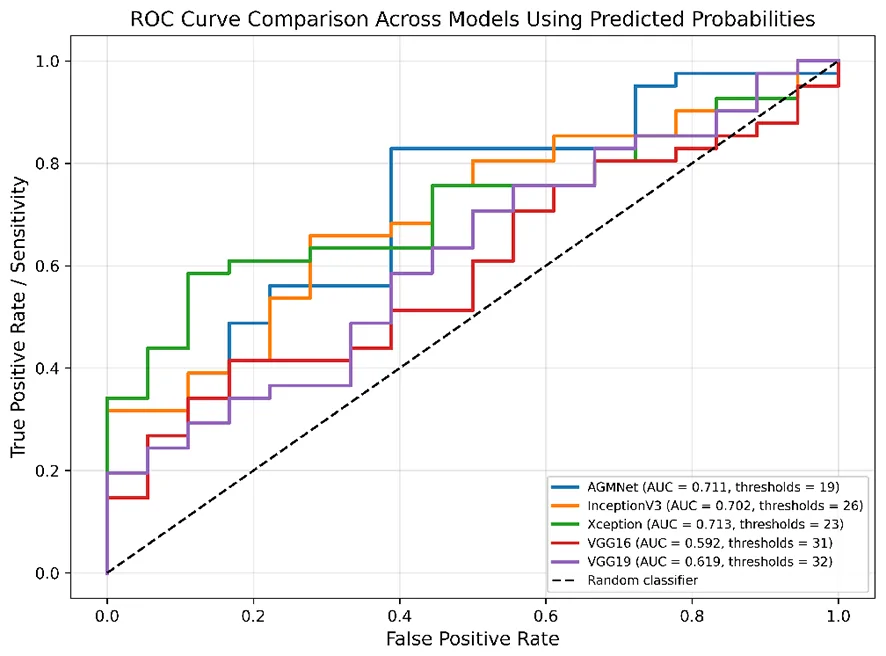
ROC curve comparison.

The ROC curve analysis shows that AGMNet consistently achieves a balance between sensitivity and specificity at the various decision thresholds, as indicated by the uniform shape of the curve, which remains above the random classifier line. While there are slight variations in AUC performance, AGMNet shows stable classification performance, which is also reflected in its better accuracy and MCC reported in Table 1.

### 5.4 Explainability analysis using grad-CAM

Figure 3 shows Grad-CAM was used to enhance interpretability on both MRI and DaT. The model emphasizes clinically relevant areas on which to make decisions. The activation in MRI images is spread out to cortical structures, which is sensitive to structural changes. The striatal area, which is a crucial biomarker in Parkinson's disease because of dopamine transporter decrease, is the focus of the DaT Grad-CAM, in contrast.This demonstrates that the suggested AGMNet model is both clinically interpretable and correct, as it is consistent with recognized neurological patterns of Parkinson's disease.

**Figure 3:**
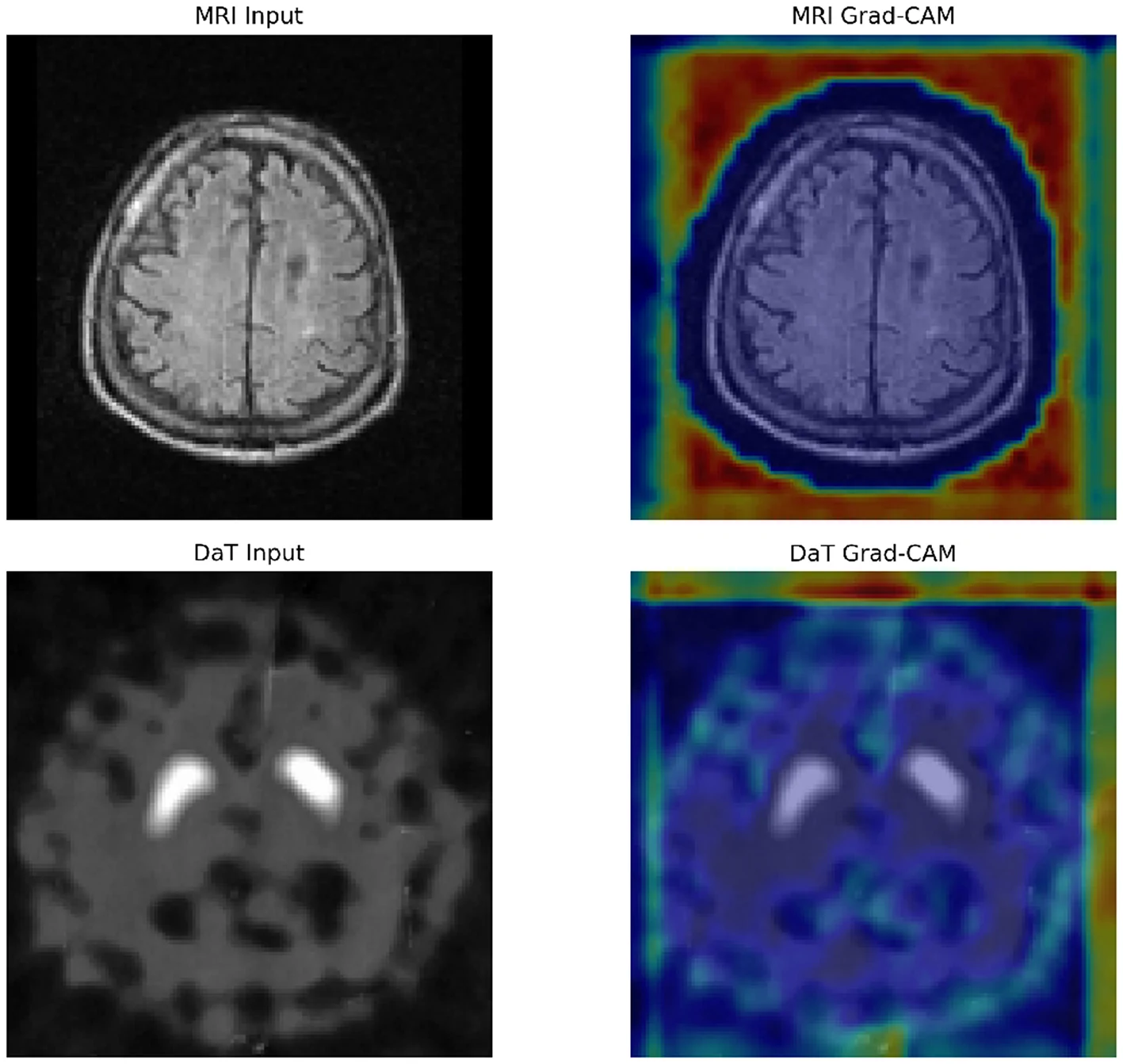
Grad-CAM visualization for MRI and DaT modalities.

### 5.5 AGMNet processing pipeline visualization

Figure 4 shows the suggested AGMNet framework step-by-step. MRI and DaT inputs, feature extraction, mean feature map creation, asymmetry computation, and gated multimodal fusion are all part of the pipeline. Each modality's relative contribution to the forecast is shown by the final gate weights. The internal operation of the suggested AGMNet framework is shown in Figure 4. Modality-specific feature extraction networks are used to process the MRI and DaT inputs at the start of the pipeline. Dominant activation patterns are then captured by aggregating the collected features into mean feature maps.

**Figure 4:**
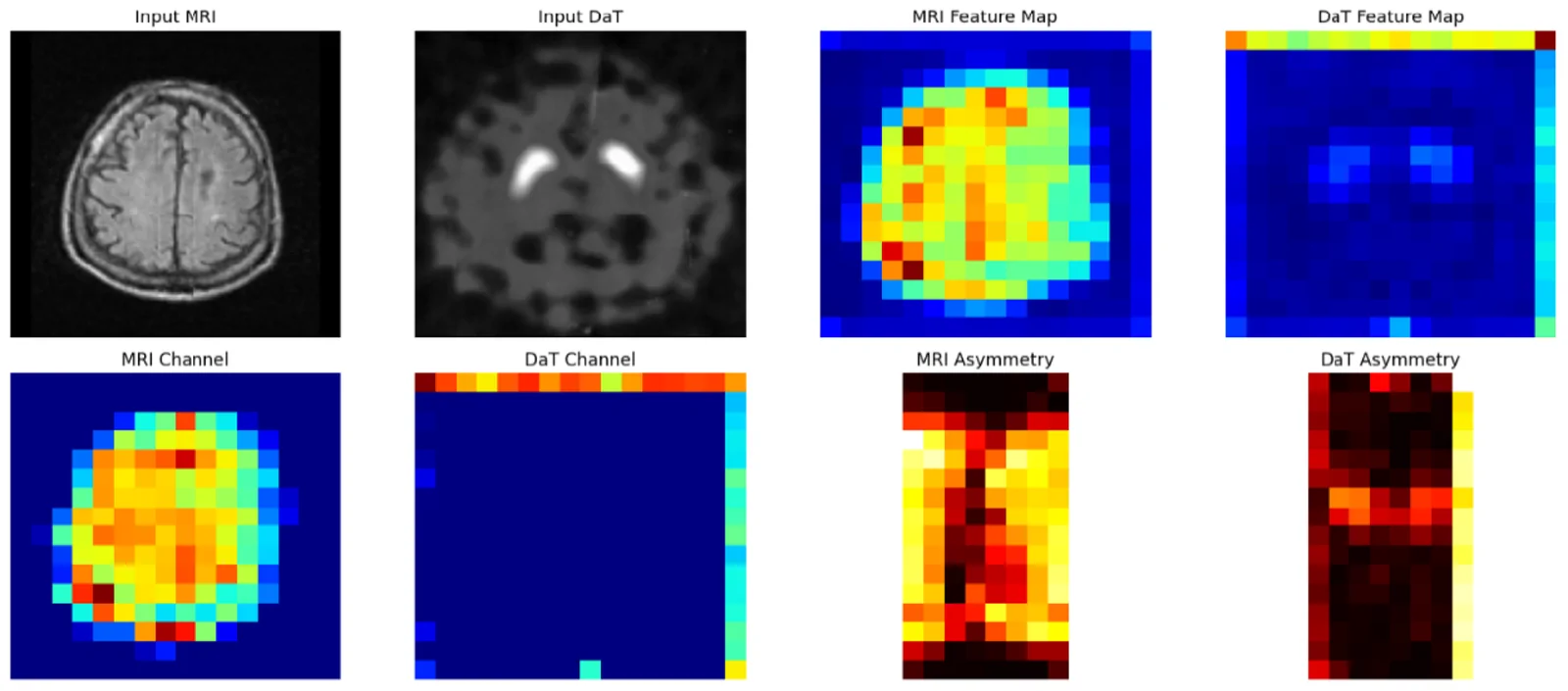
AGMNet preprocessing pipeline.

Asymmetry maps are then calculated to show the distinctions between the left and right hemispheres, which are important markers of Parkinson's disease. The model's sensitivity to lateralized neurodegeneration is increased by these asymmetry traits. Lastly, by giving each modality adaptive weights, the gated fusion process integrates MRI, DaT, asymmetry, and biomarker information. The model dynamically prioritizes various features for categorization, as seen by the gate weight visualization. Both performance and interpretability are enhanced by this organized process.

### 5.6 Subject-wise gate weight analysis

Figure 5 represents Subject-level modality contribution with gate weights. A distinct subject is represented by each subplot.

**Figure 5:**
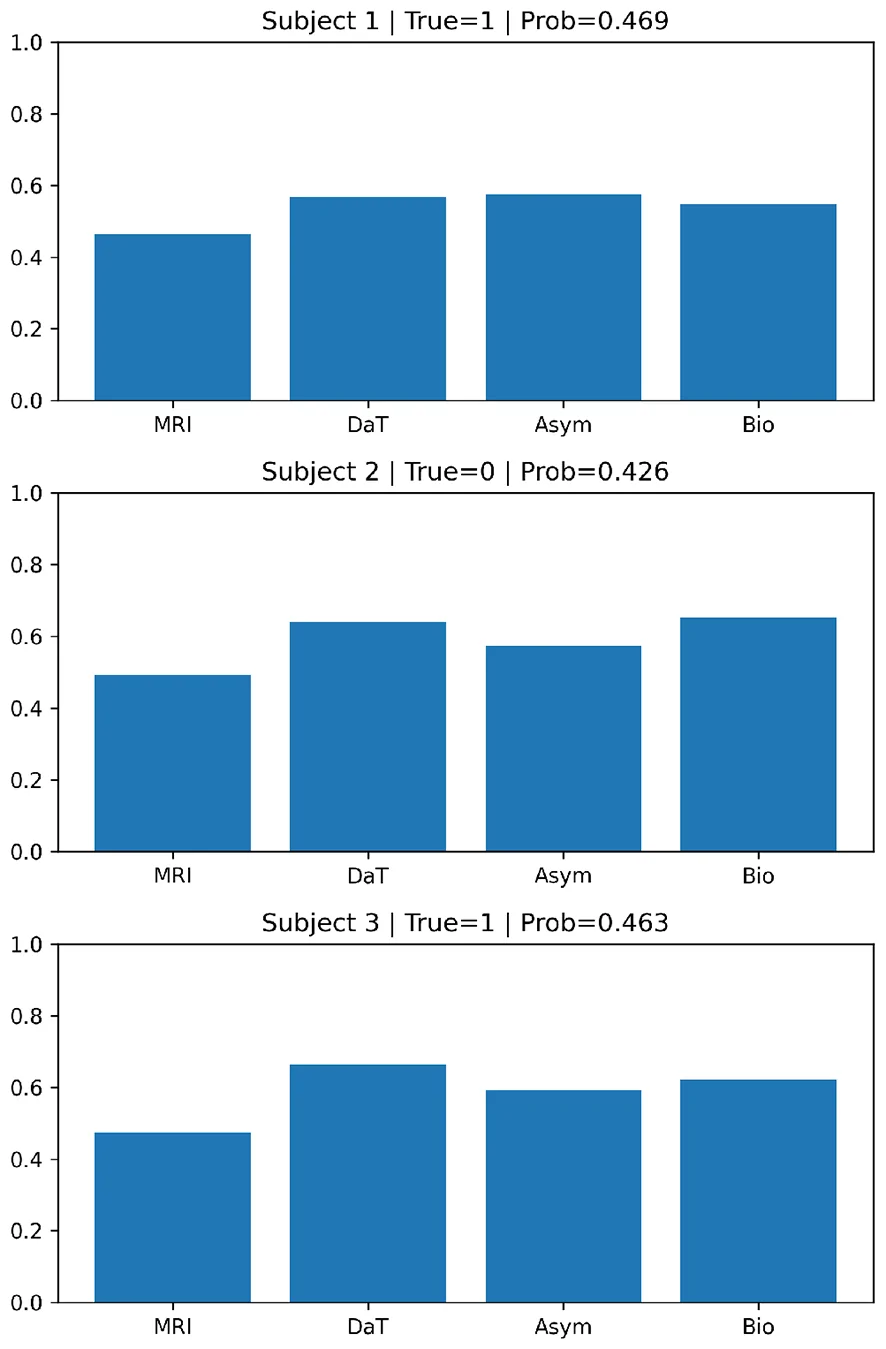
Modality contribution by subject. Note: X-axis-Modality, Y-axis-Gate Weight (dimensionless).

The gate weights allocated to various modalities for each subject are displayed in Figure 5. The findings show that biomarker characteristics, asymmetry, and DaT are given more weight than MRI.

This demonstrates that features based on asymmetry and function are more informative for the detection of Parkinson's disease. The variance across patients shows how the gated fusion mechanism is flexible, enabling customized interpretation.

### 5.7 Gate weight distribution

The average contribution of each modality is shown in Figure 6. The average weights of biomarker and DaT characteristics are greater, followed by MRI and asymmetry. This shows that integrating structural and functional data greatly enhances classification performance, supporting the AGMNet concept.

**Figure 6:**
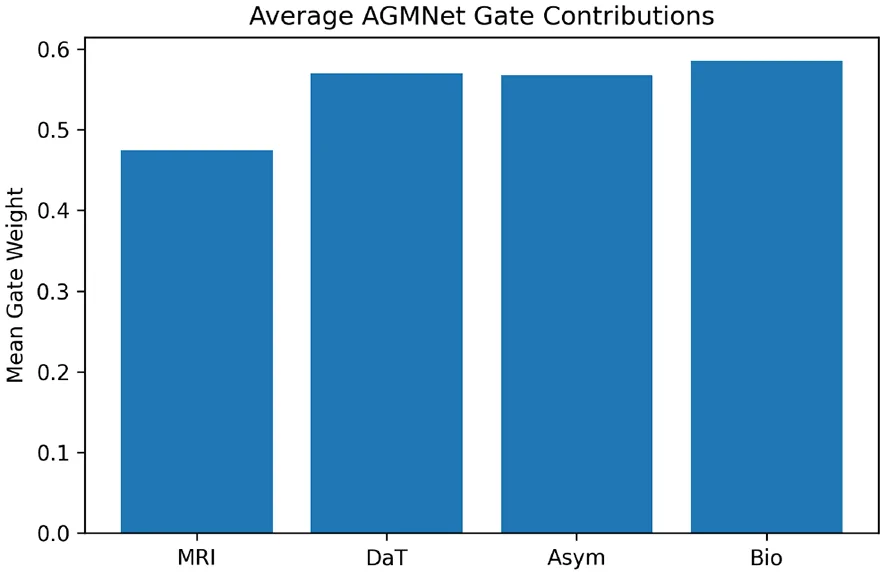
AGMNet's average gate weight distribution across modalities. Note: X-axis-Modality, Y-axis-Mean Gate Weight (dimensionless).

This study confirms that the proposed AGMNet model can be used to classify Parkinson's disease using multimodal neuroimages. The framework takes advantage of complementary structural, functional and asymmetry-aware features to better capture disease-relevant features.

### 5.8 Interpretation of performance

With respect to classification performance, AGMNet attains a mean accuracy of 0.8318 and a balanced accuracy of 0.8278, suggesting consistent results across folds. AGMNet achieves an AUC of 0.711, which indicates good predictive performance relative to state-of-the-art deep learning methods. Additionally, the Matthews Correlation Coefficient (MCC) of 0.6458 indicates a high degree of correlation between the predicted and actual classes, suggesting that the proposed model is robust.

In summary, AGMNet demonstrates a balanced sensitivity and specificity, which makes it an ideal choice for accurate classification of Parkinson's disease in multimodal cases.

### 5.9 Significance of multimodal learning

The findings also make it very clear that single-modality models cannot be used to detect Parkinson's disease with accuracy. VGG16 and VGG19 models recorded medium results with an unbalanced sensitivity to specificity ratio. Conversely, AGMNet utilizes multimodal data and performs equally in all measures of evaluation. The gated fusion mechanism allows the model to optimistically use the most informative modality to enhance the overall classification accuracy.

### 5.10 Role of asymmetry features

The proposed framework is consistent with the incorporation of descriptors that account for hemispherical differences to measure the imaging patterns. Clinically, DaT-SPECT images are typically analyzed using the pattern of dopamine transporter uptake in the striatum. In healthy subjects the uptake is relatively symmetrical and in Parkinsonian syndromes, the uptake may be reduced and hemispherical asymmetry may be present. Based on these imaging features, the asymmetry-aware descriptor was used as an auxiliary quantitative feature in the multimodal classification framework. The feature contribution analysis indicates that the asymmetry-related information can be used in the proposed fusion strategy as it improves the discriminatory information along with the structural MRI and DaT imaging features.

### 5.11 Fusion strategy comparison

In this work, we compare the proposed AGMNet against four representative multimodal fusion strategies: Early Fusion, Late Fusion, Attention-based Fusion, and Transformer-based Fusion as shown in Table 6. AGMNet had the best classification accuracy (0.8318), balanced accuracy (0.8278), and Matthews correlation coefficient (MCC) (0.6458) of all the methods evaluated, resulting in the best overall performance in classification. Conventional feature aggregation strategies—such as Early Fusion and Late Fusion—showed comparatively lower performance for all the evaluation metrics, suggesting that these are not as effective for multimodal Parkinson's disease classification. The proposed attention-based Fusion and Transformer-based Fusion methods achieved improvements over the traditional fusion techniques, especially in the aspect of ROC-AUC; nevertheless, the overall classification accuracy and MCC were still slightly inferior to the proposed AGMNet. Yet, even though its ROC-AUC was the highest of the baseline fusion strategies (0.6802), Transformer-based Fusion was still worse than AGMNet on all the reported evaluation metrics. The results show that the proposed adaptive gated fusion strategy is more effective in multimodal feature integration than the traditional fusion strategies tested in this work.

**Table 6:** Comparison of different multimodal fusion strategies.

Fusion strategy	Accuracy	**Baln. acc**.	**MCC**	ROC–AUC
Early fusion	0.6610	0.5224	0.0601	0.5867
Late fusion	0.5424	0.4993	-0.0013	0.4756
Attention fusion	0.6102	0.5481	0.0948	0.5325
Transformer fusion	0.6610	0.5535	0.1225	0.6802
**AGMNet (proposed)**	**0.8318**	**0.8278**	**0.6458**	**0.7579**

Bold Values correspond to highest metric value for each models.

### 5.12 Computational complexity analysis

A computational complexity analysis was conducted to determine the practicality of the proposed AGMNet for clinical use is shown in Table 7. AGMNet has a total of 88,933 trainable parameters, and its model size is 0.37MB, showing the lightweight architecture, which has little requirement for storage. Experiments carried out on Kaggle using NVIDIA Tesla T4 GPU. The model allocated 2,476.89 MB of GPU memory for inference (6,358.00 MB reserved by the runtime), and had an average inference latency of 13.59 ms per subject, which equates to 73.58 subjects/s inference speed. These outcomes confirm that AGMNet can efficiently achieve the balance between the computational load and diagnostic performance and make it suitable for application within computer-aided Parkinson's disease screening systems requiring low computational cost and better performance.

**Table 7:** Computational complexity analysis of the proposed AGMNet.

**Metric**	Value
Trainable parameters	88,933
Model size (MB)	0.37
GPU memory (alloc.)	2476.89 MB
GPU memory (reserved)	6358.00 MB
Inference time	13.59 ms
Throughput	73.58 subjects/s
Hardware	NVIDIA Tesla T4

### 5.13 Ablation study

An ablation study was performed by enabling different imaging modalities and architectural components of the proposed framework, presented in Table 8, and the results showed that the accuracy of classifying PD reached 75.00% when only structural MRI images were used, indicating that anatomical information alone has moderate discriminative ability for PD classification. A comparable overall accuracy achieved with the DaT-only configuration, but a balanced accuracy of 81.25% and a specificity of 100% emphasized the complementary diagnostic power of dopaminergic functional imaging. The combination of MRI and DaT modality significantly increased the classification rate to 83.33%, 87.50% and 0.7071, confirming that the combination of structural and functional neuroimaging provides complementary disease features not provided by either modality alone. The ability of the framework to discriminate was further improved by handcrafted image-derived biomarker descriptors; the ROC-AUC was improved with no loss in classification accuracy, demonstrating that the handcrafted quantitative descriptors contain complementary information for diagnosis in addition to the deep imaging representations. The importance of the proposed adaptive gated fusion mechanism was also confirmed by eliminating the gating module while retaining the rest of the architecture, which led to a decrease in balanced accuracy and MCC, thus confirming that the proposed adaptive weighting of multimodal features is beneficial for feature integration and robustness of classification in the proposed approach. The ROC-AUC increased from 0.8438 to 0.8750 when biomarker descriptors were included, but the threshold-dependent classification metrics remained unchanged. This implies that rather than altering the final binary predictions at the chosen choice threshold, the biomarkers enhanced the discriminative quality of the projected probability. Such behavior is expected given the NTUA dataset's small sample size, as slight increases in probability calibration might not always result in changes in classification accuracy or associated measures. The overall performance of the complete AGMNet was the best, with a balanced accuracy of 93.75%, an MCC of 0.8165, a sensitivity of 87.50%, and a specificity of 100%, demonstrating the effectiveness of the proposed adaptive gated multimodal fusion strategy in integrating structural MRI, DaT imaging, and quantitative biomarker descriptors. The DaT asymmetry descriptor was not assessed as a standalone branch since it is not a diagnostic tool per se, but rather included in the biomarker descriptor branch together with MRI heterogeneity and DaT uptake descriptors, which is a combination of handcrafted descriptors that provide clinically useful quantitative information. The ablation study therefore tests the whole of the hand crafted branch of the biomarker descriptor, and not one or two particular handcrafted features, as these are intended to be used as a complement to the learned deep imaging representations in the proposed AGMNet architecture.

**Table 8:** Ablation study of the proposed AGMNet.

**Model configuration**	**Acc**.	**Bal. acc**.	**AUC**	MCC	**Sens**.	**Spec**.
MRI only	0.7500	0.7500	0.8125	0.4781	0.7500	0.7500
DaT only	0.7500	0.8125	0.7813	0.5976	0.6250	1.0000
MRI + DaT	0.8333	0.8750	0.8438	0.7071	0.7500	1.0000
MRI + DaT + biomarkers	0.8333	0.8750	0.8750	0.7071	0.7500	1.0000
Without adaptive gating	0.7910	0.8625	0.7813	0.6476	0.7550	1.0000
**Proposed AGMNet**	**0.8333**	**0.9375**	**0.8750**	**0.8165**	**0.8750**	**1.0000**

Bold Values correspond to highest metric value for each models.

### 5.14 Explainability analysis

The visualizations of Grad-CAM suggest that the model mainly pays attention to the image regions in the striatal region of the DaT images, consistent with findings in previous neuroimaging studies of Parkinson's disease. The result of the model attention matching with known disease imaging regions in the brain offers further clues to the characteristics supporting model classification decisions. Moreover, the visualization can enhance the comprehension of the proposed framework by emphasizing the regions of the image that affect the model's predictions. AGMNet gives visual explanations that help to understand the classification process, compared to traditional black-box based classification methods.

### 5.15 Limitation of explainability analysis

To increase the transparency and interpretability of the proposed AGMNet framework, Grad-CAM was used to perform qualitative visualization of the image regions that are important for the model's predictions. In general, the generated activation maps were similar to those previously shown in neuroimaging studies of Parkinson's disease patients and are clinically relevant to the diagnosis of the disease. The present study, however, doesn't offer a quantitative analysis of the heatmaps produced or independent clinical validation by expert neurologists or radiologists, since the annotations of the NTUA Parkinson dataset are only available at a dataset level and not at a region or pixel level, which is needed for a quantitative assessment of explainability. Accordingly, the Grad-CAM visualizations should be regarded as qualitative evidence supporting the model's predictions rather than definitive clinical validation. Future studies will investigate the clinical reliability of the generated attention maps further by adding quantitative explainability measures (in addition to expert clinical evaluation) such as localization accuracy and overlap-based measures with annotated neuroimaging datasets.

### 5.16 Gated fusion mechanism analysis

In the adaptive gating mechanism, the model is capable of giving variable weights to the multimodal feature representations for fusion. The average gate values showed that the weights for the features derived from DaT were generally higher than those of the MRI features in the dataset analyzed. This observation indicates that functional imaging information and complementary quantitative descriptors played a significant role in the classification process. Also, the difference in gate weights in the various subjects means the model is not hardcoded with a particular weighting scheme, but adapts the weights of features to the characteristics of the individual subject. Within the proposed framework, the findings give insight into the integration of information from multiple modalities, and underscore the role of adaptive fusion in multimodal feature aggregation. This adaptive weighting strategy conceptually reflects the clinical diagnostic process, in which neurologists integrate structural MRI findings, functional DaT imaging, and complementary imaging evidence while assigning varying importance to each source according to the individual patient's presentation and diagnostic context.

### 5.17 Limitations

The data set for this study is fairly small compared to the amount of patients that may exist in larger settings, potentially reducing the generalizability of the proposed framework to larger and more diverse patient populations. The specificity (100%) of the model could be partially due to the small sample of healthy controls, and should thus be interpreted with caution until confirmed in a larger and more diverse group of controls. In addition, the proposed AGMNet framework was tested solely with one set of data. Therefore, the approach needs to be further validated in external datasets from different institutions, populations and imaging protocols, to establish robustness, reproducibility and clinical validity of the proposed approach. Moreover, there is no independent cohort that has been used to validate the proposed AGMNet. While the NTUA Parkinson set is valuable for the analysis of synchronized MRI and DaT scans appropriate for multimodal learning, the framework must be tested in other larger multicenter data sets, like the Parkinson's Progression Markers Initiative (PPMI), to further evaluate its robustness and generalizability under different imaging protocols, scanner characteristics, and patient populations. This will be taken as an important path for further research. While the proposed AGMNet holds promise, there are a number of limitations to be noted. Already mentioned that is a small dataset. Five-fold cross validation was used to increase the reliability of the performance estimates, but the small number of cases in the data set might restrict the applicability of the proposed model to other clinical data sets. Second, detailed metadata about the MRI and DaT image acquisition procedure, such as scanner type, imaging methods, or reconstruction parameters, is absent from the publicly accessible NTUA Parkinson dataset. Therefore, it was not possible to evaluate the impact of acquisition discrepancies and scanner-related variability on the suggested model. Future research will use multi-center datasets with well-documented imaging collection data to examine AGMNet's resilience. This is called scanner variation and can lead to domain shift, which can impact model performance if applied to data acquired from various clinical institutions. In addition, the current framework was tested with data from a single-center cohort, and the support of the framework in multi-center heterogeneous datasets should be tested. Future research will be conducted to validate AGMNet with larger publicly available datasets like the Parkinson's Progression Markers Initiative (PPMI) and multi-center clinical cohorts to fully assess its ability to generalize. Federated learning frameworks will further be explored to facilitate collaborative learning and training of the models across different institutions without exposing patient information, allowing for models to become more robust while maintaining data privacy. These directions are anticipated to improve the clinical utility and implementation of the proposed multimodal system for the classification of PD.

Another drawback of the proposed framework is the fact that it requires structural MRI and DaT imaging. Multimodal imaging will give complementary structural and functional information, which will help in the characterization of the disease, although access to both imaging modalities may be restricted in some health care settings, due to financial constraints, infrastructure and limited availability of nuclear medicine facilities. Furthermore, Parkinson's disease is clinically diagnosed almost entirely and advanced neuroimaging is used usually in patients with diagnostic doubt. Hence, the proposed framework should be considered as a research-oriented multimodal approach for classification and further assessment of the experimental viability, scalability and generalizability of the approach is needed to consider its clinical implementation.

The framework for the proposed AGMNet was designed and tested for binary classification (Parkinson's disease and healthy controls) based on multimodal MRI and DaT imaging. Thus, the present study is to be viewed as a proof-of-concept study to show the efficacy of adaptive multimodal feature fusion, but is not meant to be an all-purpose diagnostic tool for all Parkinsonian disorders. One of the most difficult clinical and imaging differential diagnoses in routine practice is the distinction of Parkinson's Disease from atypical parkinsonian syndromes such as PSP, MSA, CBD, and scans without evidence of dopaminergic deficit (SWEDD), which can share some clinical and imaging features. This framework did not aim to differentiate between these diagnostically difficult disorders because they were not represented in the utilized data set.

In spite of these constraints, AGMNet is designed to be a clinical decision support tool and not to supplant expert neurological evaluation. In healthcare centers where both MRI and DaT imaging are routinely collected, the framework is especially applicable to cases where there is a diagnostic uncertainty regarding parkinsonism or there is a need for further imaging to support clinical evaluation. The AGMNet integrates structural MRI, functional DaT imaging, asymmetry-aware descriptors and image-derived biomarkers in an adaptive gated fusion strategy, thus offering an objective and explainable assessment that could be used in conjunction with the interpretation of multimodal neuroimaging data by neurologists. This is why the proposed framework is not intended as an alternative diagnostic system, but rather an additional aid to enhance the diagnostic confidence in complex clinical situations. Future work involves expanding AGMNet through prospective multicenter validation with larger and more diverse numbers of patients, including those with atypical parkinsonian syndromes and those with diagnostic uncertainty, as well as integration into clinical workflow software for testing its clinical applicability and generalizability in the real world.

### 5.18 Future work

The next step of work will be to implement the proposed framework to bigger multi-center datasets to enhance generalization. The performance could be improved by the addition of other modalities like PET imaging or clinical data. Moreover, an explanation-based AI method combined with quantitative biomarkers can be used to identify Parkinson's disease early and learn more about how the condition progresses.

## 6 Conclusion

The Adaptive Gated Multimodal Network (AGMNet) is an interpretable deep learning model for PD classification with multimodal imaging. It incorporates structural magnetic resonance imaging (MRI) and functional dopamine transporter (DaT) imaging with asymmetry-sensitive features and biomarker representations via an adaptive gated fusion.

The model is evaluated in a 5-fold cross-validation experiment and it performs on par with other state-of-the-art deep learning models. AGMNet achieves an accuracy of 0.8318 and shows a high correlation between the predicted and the true labels as indicated by the MCC. While the AUC values of the models are similar, the ROC curve confirms AGMNet's consistent sensitivity-specificity trade-off across decision thresholds.

The interpretability of the proposed model is another significant strengths. Grad-CAM visualizations reveal important anatomical structures, such as the striatum in DaT scans, and the gating mechanism offers information about the contribution of each modality. Additionally, the incorporation of asymmetry-aware descriptors adds complementary characteristics to the multimodal classification framework and offers more details about hemisphere imaging disparities.

While the results are encouraging, there are some limitations. The research is limited by a smaller study population and lack of external validation, which could limit its generalizability. The next steps involve validating the model on larger and multi-site data sets and expanding the model to include other data types such as clinical data and longitudinal data.

Overall, the AGMNet framework provides a robust and explainable model for the classification of Parkinson's disease. The promise of the suggested framework for multimodal Parkinson's disease classification is demonstrated by the combination of multimodal fusion, adaptive feature weighting, and explainability mechanisms, which encourages additional validation using bigger and more varied datasets.

## Data Availability

Publicly available datasets were analyzed in this study. This data can be found here: https://www.kaggle.com/datasets/shayalvaghasiya/ntua-prakinson/data.
